# Clinical nursing application of parenteral nutrition combined with enteral nutrition support in neurosurgery

**DOI:** 10.4314/ahs.v23i3.64

**Published:** 2023-09

**Authors:** Meiying Huang, Sha Yang, Aixia Gu, Mingxia Xu, Cong Sha

**Affiliations:** Department of Nursing, The First Affiliated Hospital of Shandong First Medical University & Shandong Provincial Qianfoshan Hospital, Jinan, China

**Keywords:** Neurosurgery, parenteral nutrition support, enteral nutrition support, nursing effect

## Abstract

**Background:**

To explore the clinical nursing effect of parenteral nutrition combined with enteral nutrition support in neurosurgery.

**Methodology:**

200 neurosurgical patients were randomly divided into two groups. The time of parenteral nutrition combined with enteral nutrition support in our hospital (January 2021) was used as the cut-off point, the PN group and the PN+EN group were divided according to the cut-off point. Nutritional status, immune status, occurrence of adverse events, prognosis-related indicators were compared between the two groups.

**Results:**

Nutritional status and immune status at 7 days of nutritional support in the PN+EN group were higher than those in the PN group, The difference was statistically significant. The total incidence of adverse events in the PN+EN group (3.00%) was significantly lower than that in the PN group (11.00%), and the difference was statistically significant. The average ICU treatment time, average hospital stay and emerging infection rate in the PN+EN group were lower than those in the PN group, and the differences were statistically significant (*P* < 0.05).

**Conclusion:**

Parenteral nutrition combined with enteral nutrition support in neurosurgery can achieve a more ideal intervention effect. It is beneficial to the prognosis of patients and has a certain value of promotion and application.

## Introduction

Neurosurgery is indicated for neurological diseases such as brain and spinal cord due to trauma, including but not limited to cerebral hemorrhage, brain trauma caused by car accidents, and ruptured cerebral aneurysms^1^. Unlike other surgical conditions, patients with such conditions mostly injure the nervous system, generally have symptoms such as coma, drowsiness, dizziness, and dyspnea, require absolute bed rest, and cannot eat normally^2^. Difficulties in eating normally prevent patients from replenishing their daily nutritional requirements, which in turn may lead to a disruption of basal metabolism, resulting in a decrease in immunity, affecting the repair and compensation of the central nervous system, further aggravating the patient's condition, increasing the risk of complications and increasing the rate of death. Parenteral nutrition support is mainly used to supply the nutritional elements required by patients through intravenous route, including calories, amino acids, vitamins, electrolytes and trace elements, so as to maintain the basic nutritional status of patients^3^. However, neurosurgical patients are mostly severely ill, and the condition is characterized by variable, and mutated conditions, and the effect of single parenteral nutrition support is limited. Enteral nutrition support delivers nutrients directly into the intestine through oral administration and catheter infusion, and is directly absorbed and utilized through the intestine, which has a positive impact on improving their overall nutritional absorption status^4^,^5^. Therefore, this study was conducted to compare the effects of parenteral nutrition support and parenteral nutrition + enteral nutrition support in neurosurgery patients, with the aim of analysing the effects of this combined intervention in neurosurgery, in order to provide reference for the selection of nutrition support for neurosurgery patients.

## Patients and methods

### Patients

From January 2020 to December 2021, 200 neurosurgical patients admitted to our hospital were selected as the study subjects.

**Inclusion criteria set as follows: (**1). Patients meet the diagnosis and treatment criteria of neurosurgery6; (2). Aged ≥ 18 years old; (3). Patients could not eat normally; (4). General information was complete;5. Patients were at severe nutritional risk.

**Exclusion criteria involved as follows: (**1). Patients with diabetes, thyroid disease and other metabolic diseases, affecting the body nutrient absorption; (2). Patients combined with other serious organic diseases; (3). Patients combined with chronic enteritis, gastric ulcer and other gastrointestinal diseases; (4). Excessive condition, pre-survival ≤ 2 weeks; (5). Patients combined with other serious tumor diseases; (6). Patients with mental illness, low cooperation.

The time of development of parenteral nutrition combined with enteral nutrition support in our hospital (January 2021) was used as the cut-off point to divide them into two groups. The PN group (100 cases, January 2020 to December 2020, with conventional parenteral nutrition support) and the PN+EN group (100 cases, January 2021 to December 2021, with parenteral nutrition combined with enteral nutrition support) were divided according to the cut-off point. In PN group, there were 57 males and 43 females, aged 30 ~ 88 years old, (60.82 ± 12.79) years on average; the types of symptoms were cerebral hemorrhage disease in 56 cases, ischemic disease in 24 cases, and others in 20 cases. In PN+EN group, there were 58 males and 42 females, aged 28 ~ 96 years old, (62.85 ± 14.47) years on average, the types of symptoms were cerebral hemorrhage disease in 50 cases, cerebral ischemic disease in 26 cases, and others in 24 cases. There was no significant difference in the general data between the two groups (P > 0.05). The patient and family agreed and signed the informed consent form, which was reviewed and approved by the hospital ethics committee.

### Intervention methods

In PN group, all patients were given symptomatic support treatment after admission. Parenteral nutrition support was given within 72 h after admission, and parenteral nutrition injection (Liaoning Hesco Pharmaceutical Co., Ltd., State Drug Administration H20153094, specification 20.72 g (total amino acids)/1000 mL/bag) was administered through the subclavian vein via a deep subclavian vein tube for 24 hours. Parenteral nutrition support was given for 1 week. If the patient needs to continue parenteral nutrition after 1 week, the current nutritional regimen will be continued.

In the PN+EN group, the patients were all given symptomatic support treatment after admission, using parenteral nutrition combined with enteral nutritional support. The parenteral nutrition support in this group was the same as that in the control group, except that enteral nutrition support was added on top of it for combined intervention. The enteral nutrition support was started within 72h after the patients were admitted to the hospital. The enteral nutrition solution (Newdisia Pharmaceutical (Wuxi) Co., Ltd, State Drug Administration H20010285, specification 500mL/bottle) was given via intranasal feeding, with nitrogen amount 0.2~0.24 g/ kg/d, caloric intake around 83.68 KJ, or 1.0~1.1 times resting energy consumption, or appropriate according to the patient's specific situation dilution, the amount of nitrogen can be gradually increased to 0.24-0.48 g/kg/d and the caloric supply increased by 125.52-146.44 KJ/ kg/ or 1.5-2.0 times the resting energy consumption in the later stage. The speed should be adjusted at any time according to the patient's nutrient absorption and gastrointestinal motility. Attention should be paid to aspirating gastric residual fluid once every 3 hours to ensure gastric retention <150 mL. In addition, depending on the patient's condition and nutritional status, intravenous supplementation of vitamins, sodium ions, trace elements, and other substances should be given. This group is parenteral nutrition combined with enteral nutrition support for 1 week. If the patient needed to continue the current nutrition modality after 1 week, the current nutrition regimen was continued.

### Observation indicators

**1. Nutritional status:** At admission and 7 days after nutritional support, fasting cubital venous blood was collected from patients in the morning, centrifuged to process and separate serum, and albumin, prealbumin, hemoglobin and transferrin were detected with an automatic chemiluminescence immunoassay analyzer (model BKI1100) purchased from Boke Holding Group Co., Ltd.

**2. Body immune status :** During the two periods of admission and 7 days after nutritional support, 5 mL of fasting cubital venous blood was collected from the patients in the morning, and lymphocyte count was measured and recorded using a lymphocyte counter (model: BD FACSCountTM) purchased from BD Immunocytometry Systems; after centrifugation of the blood samples, the upper serum was separated, and immunoglobulin G and immunoglobulin M were detected and recorded using enzyme-linked immunosorbent assay kits and supporting reagents purchased from Shanghai Enzyme-linked Immunocytometry Systems Co., Ltd.

**3. Adverse events :** The number of patients with electrolyte imbalance, stress ulcer, diarrhea, abdominal distension and other symptoms after nutritional support intervention was counted.

**4. Prognostic relevant indicators:** Observe and record ICU treatment time and hospital stay of the two groups, take the mean value, and count the number of new infections after admission (including but not limited to pulmonary infection, postoperative incision infection, etc.).

### Statistical analysis

Statistical Product and Service Solutions (SPSS) 25.0 (IBM, Armonk, NY, USA) was used to process the data. Measurement data conformed to the normal distribution and were expressed as (^-^x±s). Independent sample t-test was used for inter-group comparison. Enumeration data were expressed as [case (%)].*X* ^2^ test was performed. P < 0.05 was statistically significant.

## Results

### Compare nutritional status between groups

There was no significant difference in albumin, prealbumin, hemoglobin and transferrin between the two groups at admission, (P > 0.05). The measured values of albumin, prealbumin, hemoglobin and transferrin at 7 days of nutritional support in the PN+EN group were higher than those in the PN group, and the difference was statistically significant (P < 0.05). See Table 1.

**Table 1:** Compare nutritional status between groups [x̅±s]

Group	Number of cases	Albumin (g/L)		Prealbumin (mg/L)		Hemoglobin (g/L)		Transferrin (mg/L)	

		Admission	Nutritional support 7 d	Admission	Nutritional support 7 d	Admission	Nutritional support 7 d	Admission	Nutritional support 7 d
PN+EN	100	36.12 ±	38.85 ±	208.56 ±	247.54 ±	112.43±	128.54±	1.67 ±	1.83 ±
group		4.34	4.86	21.34	23.14	7.43	7.85	0.21	0.25
PN	100	36.76 ±	32.37 ±	207.96 ±	196.54 ±	113.01±	99.96 ±	1.69 ±	1.49 ±
group		4.13	3.11	21.16	20.06	7.97	5.65	0.23	0.18
t value	-	1.068	11.231	0.200	16.653	0.532	29.550	0.642	11.037
P value	-	0.287	< 0.001	0.842	< 0.001	0.595	< 0.001	0.522	< 0.001

### Compare the body immune status between the two groups

There was no significant difference in lymphocyte count, immunoglobulin G and immunoglobulin M values at admission, between the two groups (P > 0.05). The lymphocyte count, immunoglobulin G and immunoglobulin M values at 7 days of nutritional support in the PN+EN group were higher than those in the PN group, and the differences were statistically significant (P < 0.05). See Table 2.

**Table 2:** Comparison of immune status between the two groups [x̅±s]

Group	Number of cases	Lymphocyte count (× 10 ^9^/L)		Immunoglobulin G (mg/L)		Immunoglobulin M (mg/L)	

		Admission	Nutritional support 7 d	Admission	Nutritional support 7 d	Admission	Nutritional support 7 d
PN+EN group	100	1.97 ± 0.35	2.17 ± 0.38	9.12 ± 0.43	11.88 ± 0.48	1.89 ± 0.25	2.15 ± 0.25
PN group	100	1.92 ± 0.32	1.78 ± 0.27	9.09 ± 0.41	7.14 ± 0.35	1.91 ± 0.27	1.67 ± 0.16
t value	-	1.054	8.366	0.505	79.791	0.544	16.172
P value	-	0.293	< 0.001	0.614	< 0.001	0.587	< 0.001

### Compare the occurrence of adverse events

The total incidence of adverse events in the PN+EN group (3.00%) was significantly lower than that in the PN group (11.00%), and the difference was statistically significant (P < 0.05). See Table 3.

**Table 3:** Comparison of incidence of adverse events [case (%)]

Group	Number of cases	Electrolyte disorder	Stress ulcer	Diarrhoea, abdominal distension	Total occurrence
PN+EN group	100	1 (1.00)	0 (0)	2 (2.00)	3 (3.00)
PN group	100	4 (4.00)	2 (2.00)	5 (5.00)	11 (11.00)
χ^2^ Value	-	0.821	0.505	0.592	4.916
P-value	-	0.365	0.477	0.442	0.027

### Prognostic parameters were compared between the two groups

The average ICU treatment time, average hospital stay and new infection rate in the PN+EN group were lower than those in the PN group, and the differences were statistically significant (P < 0.05). See Table 4.

**Table 4:** Prognostic indicators (x̅±s, n) compared between the two groups

Group	Number of cases	Mean ICU stay (d)	Mean length of stay (d)	Emerging infection rate [case (%)]
PN+EN group	100	8.45 ± 1.32	19.65 ± 2.65	5 (5.00)
PN group	100	10.65 ± 1.75	23.76 ± 2.87	16 (16.00)
t/χ^2^ value	-	10.0365	10.5214	6.438
P-value	-	< 0.001	< 0.001	0.011

## Discussion

### Parenteral nutrition combined with enteral nutrition support can improve nutritional status and immune status

Neurosurgery is a branch of surgery, which is a discipline that applies unique neurosurgical research methods to study, diagnose, treat and prevent the human nervous system (such as the brain, spinal cord and peripheral nervous system) and related affiliated institutions on the basis of surgery as the main treatment in surgery^7^. In general, brain-related diseases such as subarachnoid hemorrhage, cerebral infarction, craniocerebral injury caused by car accidents, and cerebral aneurysms belong to the category of neurosurgical diagnosis and treatment^8^. Patients with such diseases are often unable to eat normally due to various factors such as surgery and diseases, and the body is in a state of high stress consumption. Once the nutrient supplementation is insufficient on any day, it may mediate the occurrence of negative nitrogen balance and hypoproteinemia, and lead to decreased immunity of the body, increase the risk of infection, further aggravate the patient's condition and affect the prognosis.

Therefore, in order to improve the nutritional health status of neurosurgical patients, parenteral nutrition combined with enteral nutrition support was applied to the clinical intervention of patients with such diseases in this study. The study data showed that the test values of albumin, prealbumin, hemoglobin, transferrin, lymphocyte count, immunoglobulin G and immunoglobulin M at 7 days of nutritional support in the PN+EN group were higher than those in the PN group (P < 0.05), suggesting that parenteral nutrition combined with enteral nutrition support can effectively improve the nutritional status and immune status of neurosurgical patients. The reasons for analysis are as follows: from a microbiological point of view, T cells, B cells, phagocytes and other immune cells are important immune defence lines in the human body and are important components of the immune system^9^. From a molecular biology point of view, nutrients such as amino acids, vitamins, and trace elements are the basic substances to maintain cellular activity, that is, theoretically, nutrients are the “raw materials” that constitute immune cells and the immune system^10^.

Insufficient supplementation of nutrients can have a direct impact on the body's immunity, resulting in decreased body immunity, increasing the risk of infection, and then aggravating the patient's condition. And vitamins, amino acids and other nutrients as an important substance to maintain cellular activity, insufficient nutrient supplementation, malnutrition, but also have a direct impact on the self-repair of tissues and organs, seriously delaying the nervous system repair time of neurosurgical patients, so neurosurgical patients need to pay attention to the scientific and effective supplementation of nutrients. Parenteral nutrition support is intravenous nutrition supply, nutrient solution is directly infused into the blood circulation system, although parenteral nutrition support basically does not require further substance digestion by the human body, but in the actual diagnosis and treatment, parenteral nutrition support people can actually absorb and obtain limited nutrients through the blood circulation system, and their overall nutritional health status maintenance effect will be affected. Enteral nutrition can deliver nutrients directly to the gastrointestinal tract through oral administration, catheter infusion and other routes, and digest and absorb them through the gastrointestinal tract, which can further improve their overall nutritional health status^11^. Therefore, with the support of parenteral nutrition and enteral nutrition combined intervention, the patients in the PN+EN group could maintain a more stable and good nutritional health state. With the improvement and improvement of patients' nutritional status and effective supplementation of daily nutrients, their immunity can also be restored, and lymphocyte count, immunoglobulin G, and immunoglobulin M levels are on the rise. Berger^12^ and other studies showed that enteral nutrition support and parenteral nutrition support given according to the patient's condition could effectively improve the patient's nutritional health status and had a positive effect on improving the body's metabolism and immune function, which was consistent with the conclusions of this study.

### Parenteral nutrition combined with enteral nutrition support can reduce the risk of adverse events

The data of this study showed that the total incidence of adverse events in the PN+EN group was significantly lower than that in the PN group (P < 0.05), suggesting that parenteral nutrition combined with enteral nutrition support was beneficial to reduce the risk of adverse events. The reasons for analysis are as follows: neurosurgery-related diseases mostly involve the nervous system and will have a direct impact on the body's endocrine and metabolic. And brain injury in neurosurgical diseases predisposes the body to a stress state of high metabolism and high decomposition, resulting in an increased demand for nutrients. Once the body needs nutrients supplement, insufficient absorption, are easy to lead to neurosurgical patients in the early stage of the disease gastrointestinal mucosal injury, and then affect the intestinal mucosal barrier function, resulting in a large number of protein loss, seriously affect the body immunity, resulting in decreased immunity, increasing the risk of stress ulcers, electrolyte imbalance and other adverse events. Within 48 hours after admission, parenteral nutrition combined with enteral nutrition support was given as early as possible to improve gastrointestinal cell activity, maintain gastrointestinal physiological function, and reduce the probability of enterogenous infection, which facilitated timely correction of malnutrition. Fuentes *et al.^13^* showed that early enteral nutrition support had a positive effect on improving the nutritional status of patients and promoting the recovery of the body. Nutrients are placed into the gastrointestinal tract through oral administration, nasogastric feeding tube and other measures to stimulate gastrointestinal peristalsis, prevent and reduce gastric mucosal injury, and avoid atrophy of intestinal lymphoid tissue at the mucosal surface barrier, which is important to reduce the risk of adverse events such as stress ulcers and diarrhea^14^. Therefore, compared with the PN group receiving parenteral nutrition support alone, the PN+EN group receiving parenteral nutrition combined with enteral nutrition support showed better overall nutritional health status and immune function improvement, which helped patients maintain a more stable and good recovery status and played an important role in reducing the risk of adverse events.

### Parenteral nutrition combined with enteral nutrition support is beneficial to prognosis

The data of this study showed that the average ICU treatment time, average hospital stay and emerging infection rate in the PN+EN group were lower than those in the PN group (P < 0.05), suggesting that parenteral nutrition combined with enteral nutrition support has a positive impact on promoting the recovery of neurosurgical patients and is conducive to the prognosis. Ye^15^ and other studies showed that enteral nutrition support can improve the nutritional index of patients, give patients life support, and have a positive impact on improving the prognosis, consistent with the conclusions of this study. The reasons for analysis are as follows: Neurosurgical patients are mostly in a state of stress and hypermetabolism at the late stage of the disease, affected by cerebral ischemia, bleeding, perfusion and other factors, the gastrointestinal mucosal barrier is susceptible to significant damage, gastrointestinal tolerance becomes worse, prone to diarrhea, abdominal pain and other symptoms. Giving parenteral nutrition combined with enteral nutrition support as early as possible is beneficial to provide the required nutrients for the repair of tissues and organs of patients' bodies, improving the acute injury in the early stage of stress in neurosurgical diseases, and is of great significance in promoting the recovery of patients' bodies. Secondly, the effective supplement of nutrients required by the body is conducive to promote the repair of nerve cells, and has a positive impact on improving brain tissue injury caused by cerebral ischemia, hypoxia and perfusion, and cellular metabolic disorders, which is conducive to the recovery of neurological function in patients and can further shorten the ICU treatment time and hospital stay of patients. Finally, on the basis of parenteral nutrition support, the implementation of enteral nutrition support can further improve its overall nutritional health status and improve the effect, can effectively supplement the nutrients required by immune cells, and plays an important role in improving the immune function of patients. With the increase of patient immunity, the emerging infection rate showed a significant downward trend.

However, as far as this study is concerned, there are still the following shortcomings: 1. the number of study samples selected is small, and there are some errors in the study results; 2. no long-term follow-up of patients has been performed, and the long-term intervention effect of parenteral nutrition combined with enteral nutrition support on neurosurgical patients has not been demonstrated; 3. neurosurgery involves a wide variety of disease types, and there are some differences in the neurological impairment and surgical treatment methods in patients with different diseases, and no group comparison has been performed for patients with different diseases, and it has not been confirmed whether the degree of neurological impairment, surgical methods and other factors will affect the effect of parenteral nutrition combined with enteral nutrition support intervention, so whether the study results have a wide range of efficacy still needs to be confirmed by further clinical exploration.

## Author contributions

Meiying Huang and Sha Yang contributed equally to this work.

## Conclusion

In summary, the application of parenteral nutrition support combined with enteral nutrition support in the clinical intervention of neurosurgical patients can improve the nutritional health status of patients, improve the body immune function, reduce the risk of adverse events, and promote the body recovery of patients, which is of great significance in shortening the treatment and length of hospital stay of patients and is worthy of clinical application.

## Limitation

This study was grouped by time points, and there are many confounding factors, such as the underlying disease of the patients, the surgical method, and the time of surgery, which may have some influence on the results of the study, and this is the limitation of this study. Therefore, further large-scale randomized clinical studies are needed to further confirm the results of this study.

## Data Availability

The datasets used and analysed during the current study are available from the corresponding author on reasonable request.
